# Can social support be improved in people with a severe mental illness? A systematic review and meta-analysis

**DOI:** 10.1007/s12144-021-02694-4

**Published:** 2022-01-31

**Authors:** Thijs Beckers, Niek Maassen, Bauke Koekkoek, Bea Tiemens, Giel Hutschemaekers

**Affiliations:** 1 MET Ggz, Primary Healthcare Department, Minister Beverstraat 3, 6042 BL Roermond, the Netherlands; 2 Research Group Social Psychiatry and Mental Health Nursing, HAN University of Applied Science, Nijmegen, the Netherlands; 3 Behavioural Science Institute, Radboud University, Nijmegen, the Netherlands; 4 Pro Persona Research, Renkum, the Netherlands; 5 Indigo, Utrecht, the Netherlands

**Keywords:** Social isolation, Psychiatry, Social work (psychiatric), Mental health recovery

## Abstract

People with a severe mental illness often have less social support than other people, yet these people need social support to face the challenges in their lives. Increasing social support could benefit the person’s recovery, but it is not clear whether interventions that aim to improve social support in people with a severe mental illness are effective. A systematic literature search and review in MEDLINE (PubMed), PsycINFO, CINAHL, Cochrane, JSTOR, IBSS, and Embase was performed. Studies were included if they had a control group and they were aimed at improving social support in people with a severe mental illness who were receiving outpatient treatment. Summary data were extracted from the research papers and compared in a meta-analysis by converting outcomes to effect sizes (Hedges’s g). Eight studies (total *n* = 1538) that evaluated ten different interventions met the inclusion criteria. All but one of these studies was of sufficient quality to be included in the review. The studies that were included in the meta-analysis had a combined effect size of 0.17 (confidence interval: 0.02 to 0.32), indicating a small or no effect for the interventions that were evaluated. A subgroup analysis of more personalized studies showed a combined effect size of 0.35 (CI = 0.27 to 0.44), indicating a noteworthy effect for these more personalized studies. This evaluation of interventions aimed at improving social support in people with a severe mental illness suggests that these interventions in general have little or no clinical benefit. However, in a subgroup analysis the more personalized interventions have a larger effect on improving social support and merit further research.

## Introduction

It is well known that people with severe mental disorders have fewer social contacts, often of lower perceived quality, than people without such disorders. Causal mechanisms may be debated (are lower social contacts a cause or effect of severe mental disorders, or both?) but the need to increase social support in vulnerable people is often called for. Therefore, increasing social support has for long been a desired outcome of mental health care, however difficult to attain.

Although there is support for the possibility to increase social support in general (Hogan et al., 2002; Siette et al., 2017), there is currently no evidence whether it is possible to increase social support in people with Severe Mental Illness (SMI). Therefore, in this paper we start with discussing the background of the concepts of social networks and social support. Next, we outline the current knowledge on the topic of increasing social support, followed by the explaining the research question, which then leads to the methods, results, and discussion sections.

Every person needs social support in one way or another. Social support is any activity with the implicit or explicit aim to support someone that can be received from people in one or more social networks. It may, for example, help people to overcome daily hassles, make difficult choices, find comfort, etc. Although social support conceptually overlaps with the term social network, there are differences (Berkman et al., 2014; Tracy & Whittaker, 2015). Social networks describe the composition of a person’s contacts, including the quantity, the nature and the (bilateral) connections (Bruhn, 1991). These networks have most often been researched using more abstract theories and frameworks, mostly by social scientists, aiming at understanding how people relate to one another (Revenson & Gurun, 2019).

Social support on the other hand focuses on the support a person receives from his or her social network. It has multiple dimensions and influencing factors, such as emotional support, instrumental support, appraisal, and informational support (Berkman et al., 2014; Tracy & Whittaker, 2015; Wang et al., 2017). The concept of social support has usually been studied by clinical scientific disciplines, using more concrete frameworks within healthcare-oriented fields such as clinical psychology, nursing, and other health services. These studies are aimed at understanding the value that individuals have from receiving or providing social support, whether it is perceived or actually received (Bitter et al., 2020; Ducharme et al., 1994; Heitzmann & Kaplan, 1988; Melrose et al., 2015). The definition and operationalization of social support is debated across scientific fields (Bitter et al., 2020; Langford et al., 1997; Martire & Helgeson, 2017).

As such, social support has mostly been studies in clinical populations, including people with SMI. They often have less social support than other people, but the challenges these people face in life are at least equal to, and often bigger than, those of people without a SMI (Goldberg et al., 2003; Hur et al., 2019; Rogers et al., 2004). This issue of lacking social support in people with SMI has only become more worrying in the COVID-19 crisis (Wildman et al., 2021).

Increasing social support in people with a SMI could help increase their personal recovery (Corrigan & Phelan, 2004), especially when they have little social support to begin with (Hendryx et al., 2008). Additionally, social support helps people with a SMI to seek treatment for their mental health problems earlier when they need it most (Beljouw et al., 2010). People with a SMI who have better social support are less likely to have delusions (Heins et al., 2019), and they are less likely to be admitted in a psychiatric hospital, either voluntarily or involuntarily (Albert et al., 1998; van Veen et al., 2019). They also more often have paid employment (Rogers et al., 2004) and they are in better health generally (Uchino et al., 2012). Additionally, people with (severe) mental illness and insufficient support have, an increased risk for suicide (De Berardis et al, 2018; Orsolini et al, 2020). Considering these advantages of social support, increasing social support should be an important aim for people with a SMI.

In clinical practice, people with SMI report more problems with lacking social support (receiving for example practical or emotional help from other people) than with lacking social networks (meeting other people) (Corrigan & Phelan, 2004), which is why this study is aimed at social support rather than social networks. For the present study, the definition of social support of Cohen et al. (2001) was used, which has been widely utilized in both research and daily practice. Cohen et al. (2001) define social support as “social resources that persons perceive to be available or that are actually provided to them by nonprofessionals in the context of both formal support groups and informal helping relationships” (p. 129). This definition encompasses most of the points raised above, and it also provides a clear and concrete definition of social support.

However, a distinction should be made between received and perceived support when measuring social support. Received social support aims to objectively summarize the social support a person receives (often by observations), whereas perceived social support measures the type and/or amount of social support a person feels he or she has received (Uchino et al., 2012). Perceived social support therefore can be measured by a questionnaire, but is more subjective by nature (Melrose et al., 2015). Ideally, studies would evaluate both received and perceived social support to achieve a more objective measurement of social support, however received social support is complex to measure (due to the needed observations) and thus most measures of social support measure perceived social support (Berkman et al., 2014). An additional issue is the lack of measures of social support are validated for use in people with SMI (Beckers et al., 2020).

Intervention studies targeting social support as a clinical outcome are relatively scarce. In the most recent (2015) review, the authors concluded that interventions can be effective for increasing social networks in people with psychosis (Anderson et al., 2015). Although social networks are different from social support, both concepts are comparable enough to provide insight into improving social support in people with SMI. Although a considerable part of people with SMI suffer from psychosis, not all of them do. A broader approach to SMI than the 2015 review by Anderson et al. can thus be useful. Also, the 2015 review had no quality threshold for including studies. As a consequence, three of the five studies included in the review had a high or unclear risk for bias in multiple areas. Because these studies with a high or unclear risk for bias were included in the final analysis, the reliability of the review remains limited (Higgins et al., 2011). Lastly, there might have been new studies of interventions aimed at improving social support in people with a SMI.

Based on the issues outlined in the paragraphs before, we hypothesize that social support can be improved in people with SMI. This hypothesis leads us, therefore, to our research question: Are interventions aimed at improving social support effective for people with SMI in comparison to a control condition (e.g., treatment as usual or a waiting list control)?

## Materials and Methods

On 25 February 2020, the first author (T. B.) conducted a systematic literature search for studies that aimed to improve social support in people with a SMI and which used a (randomized) control group. The following databases were searched with no restrictions on date of publication: MEDLINE (PubMed), PsycINFO, CINAHL, Cochrane, JSTOR, IBSS, and Embase. The free text search included three elements (which form the inclusion criteria). *First*, either *psychiatry* or *mental health* must occur in the title or the abstract of each research paper in order to identify studies aimed at people with mental health problems. *Second*, the title must also include at least one of these terms: *social*, *network*, *person*, *personal*, *interpersonal*, *support*, or *ties* in order to identify studies that used social support as either a primary or secondary outcome. All measures of social support (either received or perceived support) are included, as long as they describe a concrete model of social support as discussed in the introduction. *Third*, the publication title or the abstract must include one of the following terms to indicate that the publication was an intervention study: *experiment*, *experimental*, *trial*, *intervention*, *program*, *training*, or *schooling* in order to identity experimental studies. Only studies where the participants met the criteria for SMI were included. SMI was defined according to Parabiaghi et al.’s (2006) definition: any mental disorder, GAF < 50 and duration of service contact < 2 years. There were no specific diagnoses in-or excluded, because although symptoms can differ between diagnoses, decreased social support is a common problem in the recovery of people with SMI (Corrigan & Phelan, 2004). Further information on the exact search strings for each database can be found in the supplementary material at the journal’s website. Search terms were deliberately broad so as to include as many relevant studies as possible. In addition to the structured search, the reference lists in the studies included in the final list of studies were hand searched for additional studies that might be relevant but which were not identified in the principal search. Finally, a CoCites search was conducted for scientific articles that were frequently cited together with the studies included in the final list (Janssens & Gwinn, 2015; Janssens et al, 2020). This was done in order to identify studies that may have been overlooked before. The study was conducted according to the most recent guidelines (Higgins et al, 2019), and it is reported according to the Preferred Reporting Items for Systematic Reviews and Meta-Analyses (PRISMA) checklist (Moher, 2009).

### Exclusion Criteria

The references that were identified in the literature search were imported into Covidence (online systematic review management software) for deduplication. Two of the authors (T.B. and N.M.) independently screened the references and abstracts for the inclusion criteria outlined before and for five exclusion criteria. First, studies in which the participants were admitted to a psychiatric hospital were excluded, because improving a social network from the restrictions of a psychiatric hospital might require a different intervention than improving the social network of a person living at home. Second, studies that lacked a control group that received routine treatment were excluded because these studies provide only limited evidence for the effectiveness of an intervention. Third, any study that did not include a measure of social support as an outcome measure (of either the quality or the quantity of the support) was excluded, because social support was the main outcome of interest. Studies that measured a derived measure of social support, for example loneliness or social skills, were excluded because there are additional factors that influence these derived measures, thus limiting their validity. Fifth and last, publications in a language other than English, German, or Dutch were excluded because these are the languages the authors can read and understand sufficiently. When a difference occurred in the assessment of the two authors (T.B. and N.M.), the study was discussed until a consensus had been reached. In the next stage, a full-text review was conducted using the same exclusion criteria and procedures as presented here.

### Data Extraction

Each of the two authors (T.B. and N.M.) independently extracted the following information from each study: Country in which the study was conducted, study setting, number of participants (intervention and control groups), inclusion criteria, intervention outline, control group details, duration of the intervention, duration of the follow-up and outcome assessments.

### Risk of Bias Assessment

All of the studies that were included were systematically assessed for risk of bias using the revised Cochrane risk-of-bias tool for randomized trials (RoB 2) (Higgins et al., 2011) or the Risk of Bias in Non-Randomized Studies of Interventions (ROBINS-I) tool (Sterne et al., 2016). The two authors (T.B. and N.M.) independently performed these assessments. Differences between their assessments were discussed until a consensus had been reached.

### Quantitative Data Extraction and Statistical Analysis

For analysis of the quantitative data, we compiled scores for the outcome measures for both the intervention and the control groups. Effect sizes were also included when they were available. Meta-essentials (a software package for meta-analysis) was used (a) to calculate Hedges’s *g* effect size and confidence intervals for all of the studies, and (b) to perform a publication bias analysis (van Suurmond et al., 2017). The DistillerSR Forest Plot Generator (Evidence Partners) was used to generate the forest plots. Three subgroup analyses were conducted to compare: (a) generic vs personalized interventions, (b) shorter vs longer follow-up periods, and (c) shorter vs longer intervention periods.

### Ethical Considerations

Ethical approval was not required because only existing and already published data were used.

## Results

### Selection of Studies

**Fig. 1:**
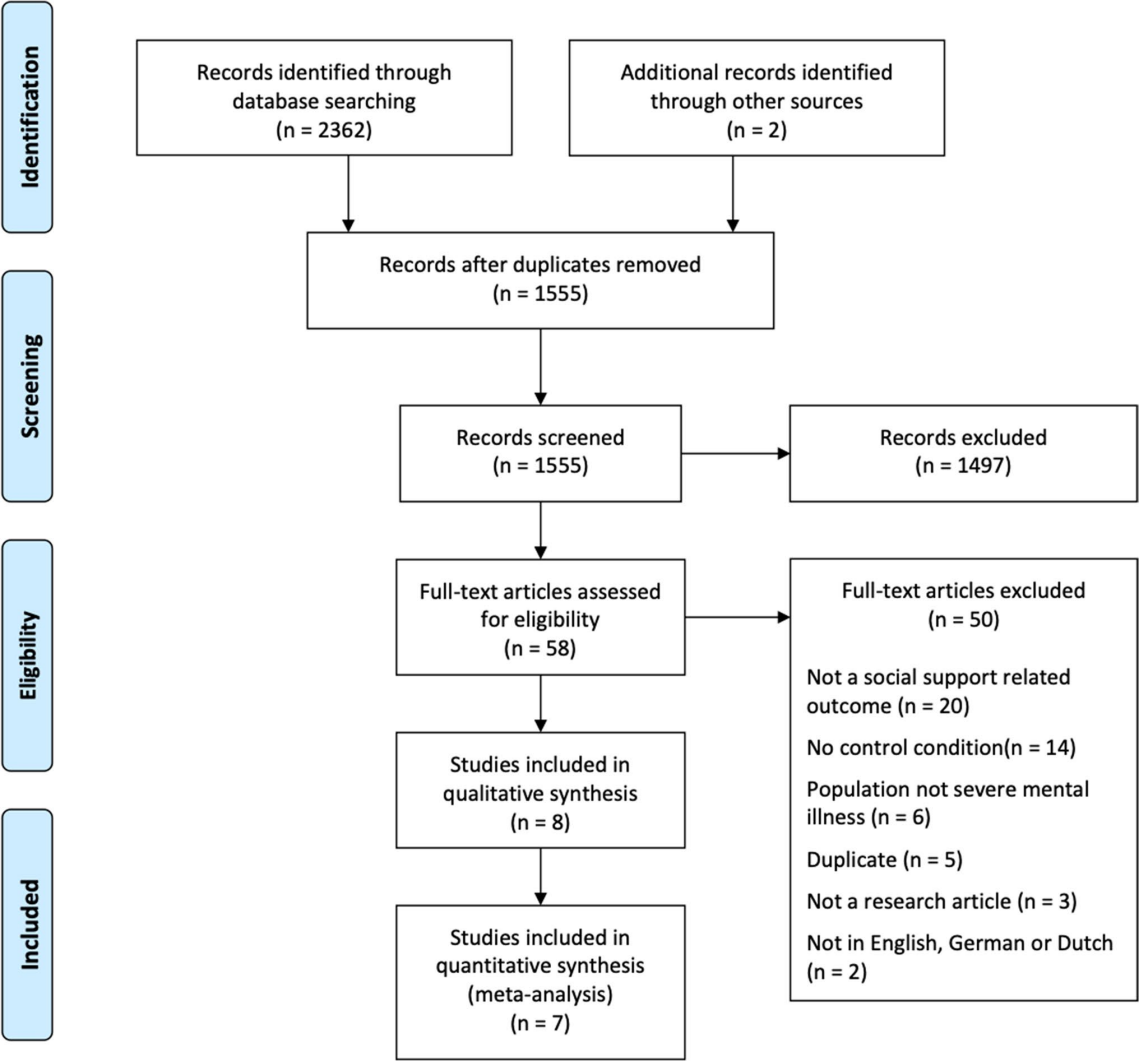
A PRISMA flowchart of the study design

The systematic search yielded 2,362 publications. Two records were identified through other sources. Of these records, 809 were duplicates (see Fig. 1). After the titles and abstracts had been screened, 58 records remained for assessment eligibility, eight of which remained after the full-text screening. Of the 50 studies that were ineligible, most of them did not meet the inclusion criteria because they did not have an outcome assessment that was related to social support; often some variant of a social skills or a social cognition questionnaire had been given instead. Other studies did not meet the inclusion criterion that required studies to have an experimental design, e.g., a control group was not included. No studies had to be excluded because of unclear definition of the target population. After the study selection process was completed, eight studies remained for inclusion in the review (Castelein et al., 2008; Davidson et al., 2004; Hengartner et al., 2016; Kaplan et al., 2011; Lecomte et al., 2008; Priebe et al., 2019; Sheridan et al., 2014; Terzian et al., 2013). All of these studies were a randomized controlled trial, even though the inclusion criteria required only experimental and control groups and not necessarily randomization.

### Study Characteristics

In most respects, the studies that were included differed considerably from one another. The sample size ranged from 107 to 345 participants, and the eight studies were performed in seven different countries, all of which were in Europe or North America (see Table 1). Two studies had two different intervention groups. The nature of the interventions that were delivered varied among the eight studies, but they could be divided into generic versus personalized interventions. Six studies used a clearly defined, manualized intervention with a specific approach, which can be classified as a generic intervention. These generic interventions included peer support groups (Castelein et al., 2008), befriending (Davidson et al., 2004; Priebe et al., 2019; Sheridan et al., 2014), Internet peer support facilities (Kaplan et al., 2011) or group cognitive-behavioral therapy or group social skills training (Lecomte et al., 2008). The remaining two studies had a more personalized intervention, in which (a) a healthcare professional visited participants along with their friends and relatives to discuss social support (Terzian et al., 2013), or (b) a healthcare professional discussed the participant’s need for social support and interaction outside of the mental health service, and he or she facilitated fulfillment of this need (Terzian et al., 2013).

**Table 1:** Characteristics of the studies included in the review

Study	Country	N (int/ control)	Inclusion criteria	Intervention outline	Control	Duration of intervention	Follow-up	Outcome measurement (questionnaire)	Details on outcome measurement
Castelein et al., 2008	Netherlands	56/50	schizophrenia or a related psychotic disorder without known drug/alcohol dependency	Closed peer support groups, guided by nurses,16 biweekly 90 min. sessions	TAU + waiting list for intervention	8 months	8 months	Social support list	Measures positive interactions and discrepancies between wanted and received support (self-rating)
Davidson et al., 2004	United States	95/95/70	people with psychiatric disabilities who were socially isolated and withdrawn	Two versions of befriending: Matched with other mental healthcare user + stipend, matched with healthy volunteer + stipend	Only stipend	9 months	9 months	Social Functioning Scale	Measures participants social engagement as well as competence in social interactions (self-rating)
Hengartner et al., 2016	Switserland	82/85	Admission in mental health hospital at moment of inclusion + GAF < 60	Visists from social worker before and after discharge from mental health hospital + care review meeting, crisis plan, involving support system	TAU	3 months	12 months	Fragebogen zur sozialen Unterstützung – Kurzform 14	Measures emotional support, instrumental support, and social integration (self-rating)
Kaplan et al., 2011	United States	101/99/100	Diagnosis in schizophrenia spectrum or affective disorder	Two experimental unstructured internet peer support interventions: listserv (anonymousl group distribution email list), bulletin board	TAU + Waiting list for intervention	12 months	12 months	medical outcomes study (MOS) social support survey	Measures functional social support in four subscales: emotional/informational, tangible, affectional and positive social interaction (self-rating
Lecomte et al., 2008	Canada	39/40/50	Recent onset psychosis (mean 4 years ill)	Two psychological interventions: group CBT or individual social skills training for symptom management	TAU + waiting list for intervention	3 months	15 months	Social Provision Scale	Measures social support in six areas: attachment, social integration, reassur- ance of worth, material support, guidance, and opportunity for nurturance (self-rating)
Priebe et al., 2019	United Kingdom	63/61	Schizophrenia or related disorder	Befriending: matching with trained volunteers	TAU	12 months	18 months	Social Contacts Assessment	social contacts defined as the number of different people met across the past 4 days (self-report)
Sheridan et al., 2014	Ireland	52/55	Enduring mental Illness	Befriending: matching with volunteers + stipend	TAU	9 months	10 months	Social Functioning Scale	Measures participants social engagement as well as competence in social interactions (self-rating)
Terzian et al., 2013	Italy	173/172	Schizophrenia or related disorder	Staff discusses social needs outside of the reach of the service with the participant and assists in achieving this activity	TAU	3–6 months	2 years	Convinience list	Scoring the participants’ reported relationships, their quality and their durarion (self-report)

The studies included in the analysis also differed in the duration of the intervention that was delivered and the length of the follow-up period. In both cases, the studies were divided into two approximately equal groups (viz., shorter vs. longer interventions and shorter vs. longer follow-up periods) based on the number of participants and the weight in the meta-analysis. The duration of the intervention ranged from 3 to 12 months, and three of the studies had shorter interventions (3-to-6 months), and the remaining five studies had longer interventions (8-to-12 months). Five of the studies had a short follow-up period (8-to-12 months), and the other three studies had a longer follow-up period (15-to-24 months). Three studies followed the participants only for the duration of the intervention. It is also noteworthy that most of the studies with shorter intervention periods had longer follow-up periods.

In most of the studies, questionnaires that had been developed to measure social support were administered. They included, for example, a list of behaviors that indicated social support or a scale that measured social functioning. Two of the studies used a list of social support behaviors that the authors themselves had developed but had not validated. These were aimed mostly at quantifying the number of people from whom the participants had received social support. None of the used measures of social support were sufficiently validated for use in people with SMI.

#### Risk of Bias Assessment in Individual Studies

The risk of bias assessment revealed that most of the studies had a high or unclear bias in only one area. One study (Sheridan et al., 2014), however, had a high or unclear risk of bias in three of the five areas that were assessed and thus was excluded from further analysis. Thus, seven studies with in total nine interventions were included in the meta-analysis Table 2.

**Table 2:** Results of the risk of bias assessment

Study	Randomisation Process	Deviation from Intended intervention	Missing Outcome Data	Measurement Of the outcome	Selection Of the Reported results	Overall
Castelein 2008	Low risk	Low risk	Low risk	Low risk	Low risk	Low risk
Davidson 2004	Low risk	Low risk	Unclear risk	Low risk	Low risk	Low risk
Hengartner 2016	Low risk	Unclear risk	Low risk	Low risk	Low risk	Low risk
Kaplan 2011	High risk	Low risk	Low risk	Low risk	Low risk	Low risk
Lecomte 2008	High risk	Low risk	Low risk	Low risk	Low risk	Low risk
Priebe 2019	Low risk	Low risk	Low risk	Low risk	Low risk	Low risk
Sheridan 2015	High risk	Unclear risk	High risk	Low risk	Low risk	High risk
Terzian 2013	Low risk	Low risk	Low risk	High risk	Low risk	Low risk

### Meta-Analysis

**Fig. 2:**
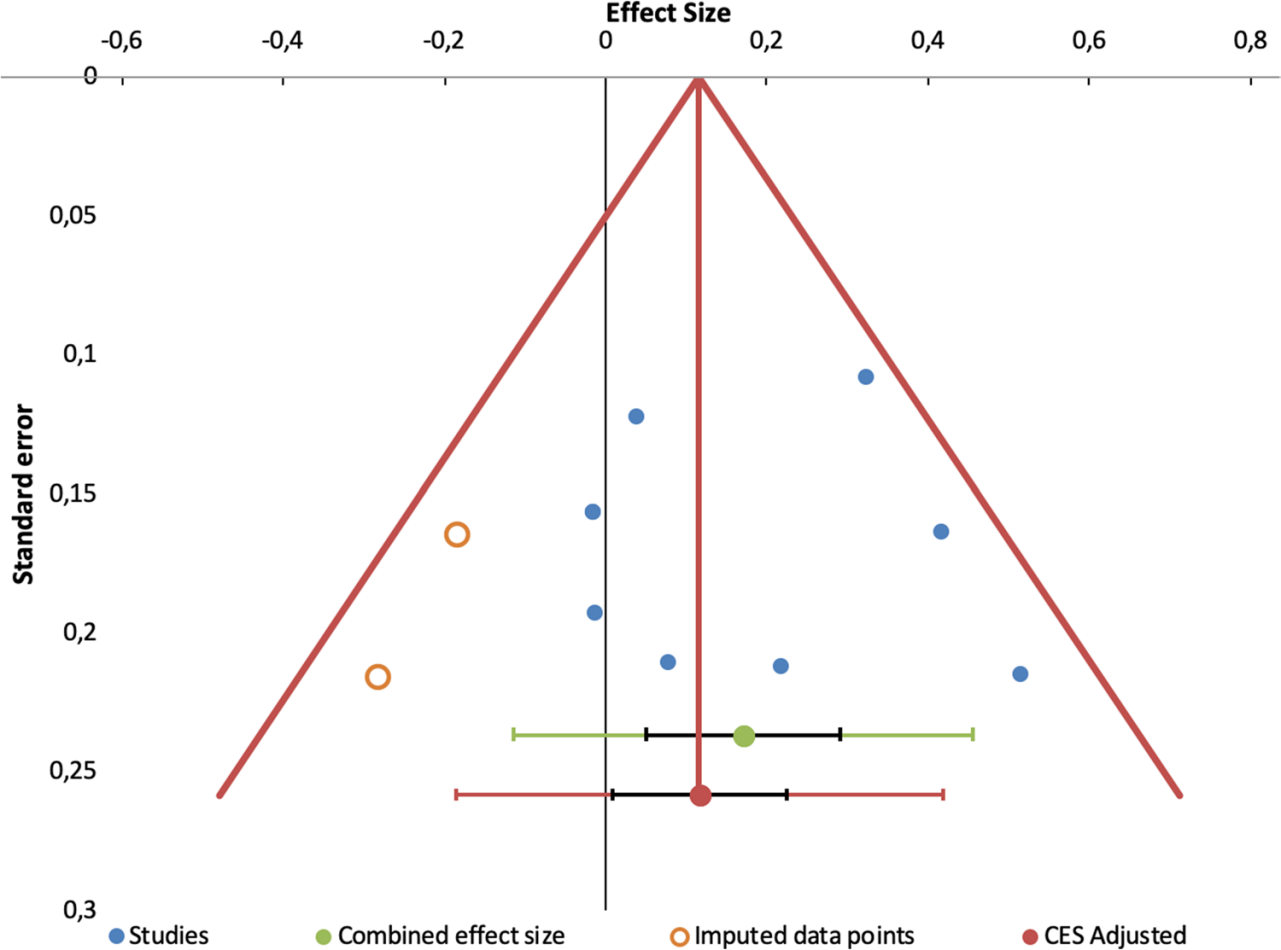
Results from an analysis of publication bias (funnel plot)

Publication bias analysis showed no indication for unpublished studies on the subject of improving social support in people with SMI (see Fig. 2).

**Fig. 3:**
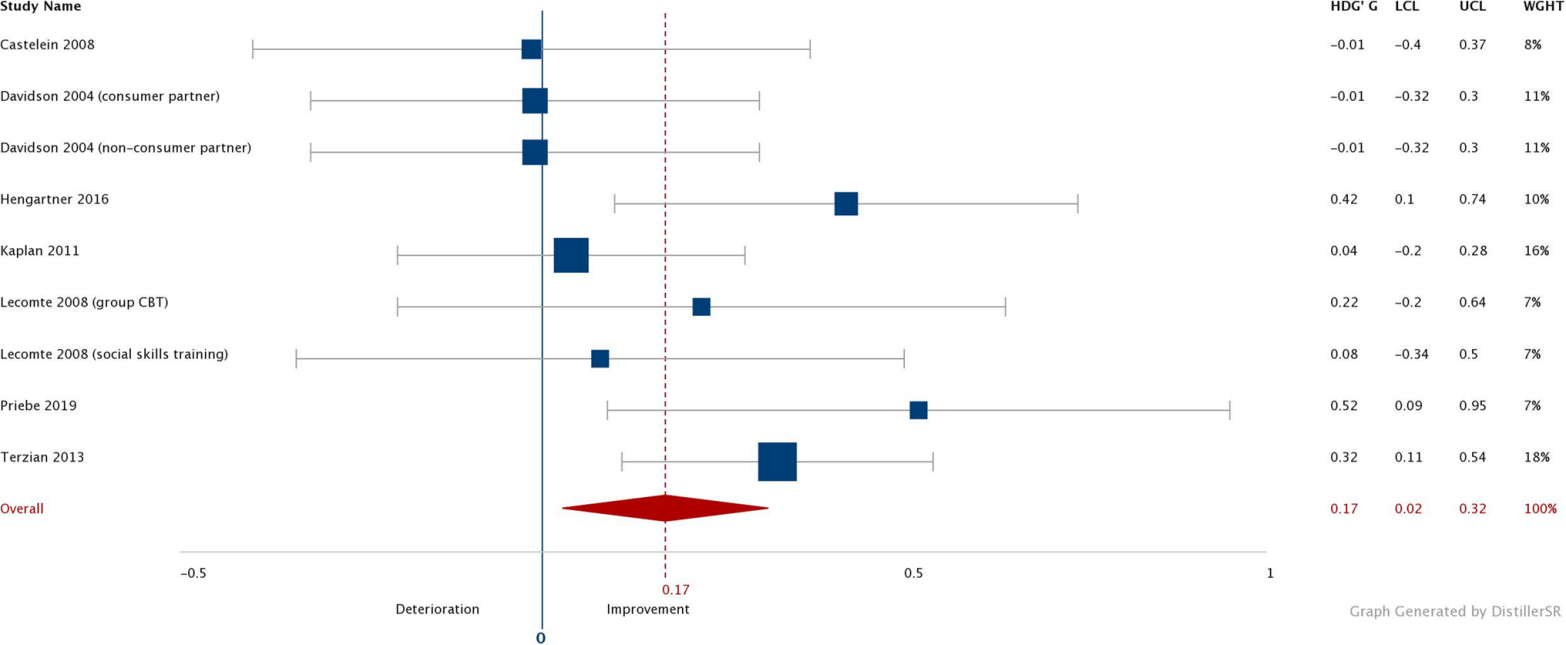
Results of an analysis to show whether social support had improved

The studies that were included in the analysis had a combined Hedges’s *g* effect size of 0.17 and a confidence interval between 0.02 and 0.32 (see Fig. 3). More than half of the studies had an effect size of less than 0.1, but some outliers had a larger effect size. Overall, the combined effect sizes indicated a small or no effect for the interventions that were evaluated.

### Subgroup Analysis

#### Generic vs. Personalized Interventions

As described earlier, two of the studies evaluated a more personalized intervention (Hengartner et al., 2016; Terzian et al., 2013). When these studies were analyzed as a subgroup, they had a combined Hedges’s *g* effect size of 0.35 (CI = 0.27 to 0.44), which is considerably higher than the combined effect size for all or the studies that were included. The generic interventions had a combined Hedges’s *g* effect size of 0.09 (CI = -0.02 to 0.20).

#### Shorter vs. Longer Follow-up Periods

As indicated in the description of the study characteristics, there were four studies with a longer follow-up period (Hengartner et al., 2016; Kaplan et al., 2011; Priebe et al., 2019; Terzian et al., 2013). Assessment of these studies with a longer follow-up period (15 months or longer) indicated a combined Hedges’s *g* effect size of 0.29 (CI = 0.09 to 0.49), compared to 0.05 (CI = -0.03 to 0.13) for the studies with shorter follow-up periods.

#### Shorter vs. Longer Intervention Periods

There were three studies with a shorter intervention period (Hengartner et al., 2016; Lecomte et al., 2008; Terzian et al., 2013). For the studies with an intervention period of six months or less, the combined Hedges’s *g* effect size was 0.30 (CI = 0.18 to 0.42) compared to 0.07 (CI = -0.07 to 0.22) for the studies with an intervention period longer than six months. The two studies that had a more personalized intervention (Hengartner et al., 2016; Terzian et al., 2013) were included both in the studies with a short intervention and in the studies with a long follow-up period. The majority of the weight in the meta-analysis (55%—74%) comes from these studies.

## Discussion

The quality of the eight studies included in this review was generally satisfactory. There was, however, a concern that most of the studies had a high risk of bias in one category. The most frequent concern was that the risk of bias during the randomization process occurred because the researchers themselves performed randomization rather than having it done independently. In short, the quality of the studies was not optimal, but it was sufficient for a thorough review and meta-analysis to be conducted.

The meta-analyses indicated that the interventions, which were aimed at improving social support in people with a SMI, had a combined effect size of 0.17. There were some outliers with higher effect sizes, but most of the studies had an effect size that was less than 0.1. In short, both the combined effect size and a substantial portion of the effect sizes of the individual studies were well below the threshold required for an effective intervention (Brooks et al., 2014). Since all studies used self-reported questionnaires to measure perceived social support, these small effect sizes suggest that the interventions aimed at improving social support in people with SMI generally do not increase the social support perceived by the participants and thus these interventions as a group have no actual benefit.

The fact that currently there is little or no evidence for the effectiveness of these interventions in general does not necessarily mean that they have no merit. There are, in fact, indications that some interventions can yield higher effect sizes and thus increase the (perceived) social support of people with SMI. Consider, for example, generic vs. personalized interventions. Generic interventions impose a strict regime on people with a SMI in order to improve their social support. Regardless of whether (a) the intervention is a structured group program, (b) the people with SMI are matched with one another or with volunteers, or (c) the support provided is Internet-based, the participants in these interventions do not have much of an opportunity to match the components of the intervention with their individual needs. Consequently, the social support that is being offered on a group level does not benefit most people with SMI and there is no improvement in social support on a group level.

Two of the studies did offer a more personalized intervention, whereby the healthcare professionals held discussions with the participants and their social network, with an aim of improving the social support. This distinction between generic and personalized interventions for people with a SMI has previously been made, and it was suggested that the distinction warranted future research (Lederman et al., 2019; Lloyd-Evans et al., 2014). In Hengartner et al.’s study (2016), patients who were being discharged from a psychiatric hospital were offered counseling together with the members of their social network, with an aim of preventing readmission. Although the readmission rate hardly decreased, social support did improve considerably. In Terzian et al.’s study (Terzian et al., 2013), healthcare professionals discussed participants’ need for social support outside the mental health service, and subsequently they worked for three months with the participants to help them achieve their goals. The two studies had a combined effect size of 0.35, which was still small but well above the effect size obtained with the other interventions and in line with other psychosocial interventions (Brooks et al., 2014). Although the present subgroup analysis contained only two studies, the studies did represent 28% of the weight in the meta-analysis as a whole. The effect sizes in these two studies were close to each other and noticeably higher than the effect sizes in the other studies, thus underlining the potential of the more personalized interventions.

The interventions in the studies with a longer follow-up period (15 months or longer) had a small effect on the social support of people with a severe mental illness, whereas the studies with a shorter follow-up period showed no effect. This is noteworthy because having a follow-up period that extends considerably beyond the end of the intervention is essential to adequately assess the risks and benefits of the intervention that is being assessed (Llewellyn-Bennett et al., 2018). The fact that a larger effect was obtained in the studies with a longer follow-up period also suggests that being able to improve social support takes time, although this effect is not certain because the confidence intervals of both groups have some overlap. We suggest, however, that having an additional follow-up period that extends considerably beyond the end of the customary follow-up period would be important when an intervention aimed at improving social support in people with a SMI is being evaluated.

Anderson et al.’s (2015) earlier review and meta-analysis of studies aimed at improving the social network of people with a psychosis has much in common with the present study, but it differs in three notable ways. First, Anderson et al. (2015) included only studies whose participants were people with a psychotic disorder, whereas our study included studies whose participants were people with any kind of SMI. Second, Anderson et al.’s (2015) analysis included studies that had used more theoretical outcome measures, such as social cognition, whereas our study included only outcome measures that were directly related to social support. Third, Anderson et al. (2015) included studies with multiple high or unclear risks for bias, whereas we excluded studies with a high or unclear risk of bias in more than one area. Because of these differences, only two trials were included in both the present study and in Anderson’s study, resulting in the two studies arriving at different conclusions. Specifically, Anderson et al. (2015) concluded that interventions directly targeting social isolation in general can be effective in improving the social network of people with a severe mental illness. We, on the other hand, were more reserved in our conclusions and conclude that the more personalized interventions specifically can be effective in improving the social support of people with a severe mental illness.

The strong points of the current study are the rigorous methods used in the review and the meta-analysis. Also, the results are reported according to state-of-the-art guidelines. One potential weakness is that the social support part of the search string was only aimed at the title, which might increase the risk of not finding studies that used social support as a secondary outcome. However, the secondary search strategy of using a CoCites search (with the eight included studies as input) to identify studies on the same topic the primary search strategy missed did not yield additional results, which indicates there probably are no relevant studies missed by the primary search strategy.

When considering the primary studies on which this review is based, the risk of bias in the studies that were included was limited; thus, these studies form a firm basis for the review and the meta-analysis. One concern is that seven different measures of social support were used in the eight studies. This is especially problematic because none of the measures has been sufficiently validated for use with people with a SMI, which is especially important when social support is being measured (Beckers et al., 2020; Bruhn, 1991).

In conclusion, in this evaluation of interventions aimed at improving social support in people with a SMI, showed a statistically significant, but not clinically relevant effect on the perceived social support. However, there is a noteworthy difference between the generic and more personalized interventions, where the more personalized interventions had a larger and clinically relevant effect on improving social support. Future research should be directed at improving and evaluating personalized interventions aimed at improving social support in people with a SMI, and should include an extended follow-up evaluation.

## Data Availability

All data generated or analysed during this study are included in this published article or are available from the original studies included in this article.
